# Reading chest radiographs in the critically ill (Part I): Normal chest radiographic appearance, instrumentation and complications from instrumentation

**DOI:** 10.4103/1817-1737.49416

**Published:** 2009

**Authors:** Ali Nawaz Khan, Hamdan Al-Jahdali, Sarah Al-Ghanem, Alaa Gouda

**Affiliations:** *Department of Medicine and Medical Imaging, King Saud University for Health Science, King Abdulaziz Medical City, King Fahad National Guard Hospital, Riyadh, Saudi Arabia*

Interpreting chest radiographs of the critically ill patients who are in intensive care units (ICUs) poses a challenge not only for the intensive care physicians but also for the radiologist. These challenges arise because of several factors:[1] ICU patients are prone to several cardiopulmonary disorders which when superimposed on the underlying pathology that prompted admission create a complex radiological appearance, which may be difficult to interpret on the basis of imaging findings alone.[2] The standard posteroanterior (PA) radiograph is replaced by the suboptimal anteroposterior (AP) radiograph in the ICU patient.[3] Instrumentation, mechanical ventilation, cardiac and other vital sign monitoring and feeding tubes, etc., detract from other findings on the ICU chest radiograph.[4] Radiologists/Intensive care physicians are under pressure for rapid interpretation of chest x-rays when treating critically ill patients, often with inadequate clinical information, partly due to the fact that things can change rapidly in the critically ill.[5] Radiological interpretation is hampered by the bewildering array of line placements in the ICU patient, where incorrect placement is not uncommon, which may not be obvious to the observer without clinical input.[6] Air space shadowing in the ICU patient may have identical appearances in a variety of cardiopulmonary pathologies. Although the imaging modality of choice in the ICU patient remains that of chest radiography, computed tomography is often performed as computed tomographic pulmonary angiography (CTPA) with suspected pulmonary embolism. Ultrasound is used to confirm pleural and pericardial effusions and when pleural intervention is planned.

The aim of this paper is[1] to discuss the radiographic findings of cardiopulmonary disorders common in the ICU patient and suggest guidelines for interpretation based not only on the chest radiograph but also on the pathophysiology and clinical grounds.[2] In addition, to describe the normal position of monitoring devices and other line placements, and prompt recognition when they are misplaced or when other complications occur.

This is a 2-part series[1]: Part I: Normal chest radiographic appearances in the ICU patient, correct and incorrect placement of various intra-thoracic tubes and lines and complications from instrumentation.

Part II: Radiography of lung pathologies common in the ICU patient.

## The Normal Chest X-ray

**Figure 1:**
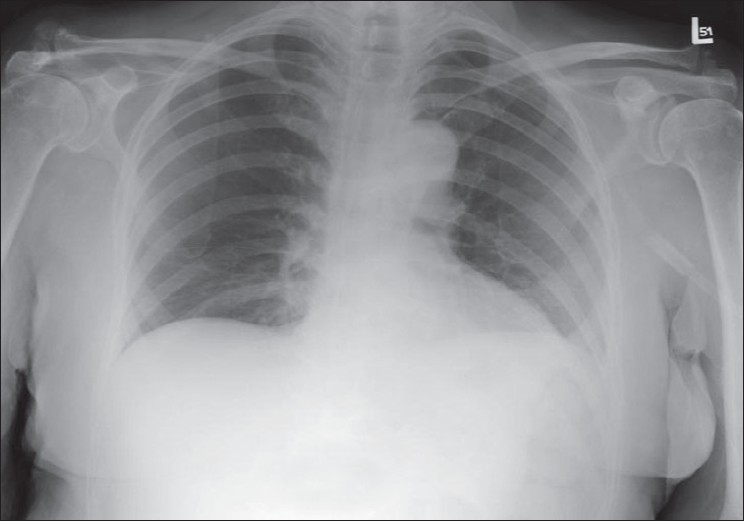
A normal AP chest radiograph of an ICU patient

**Figure 2:**
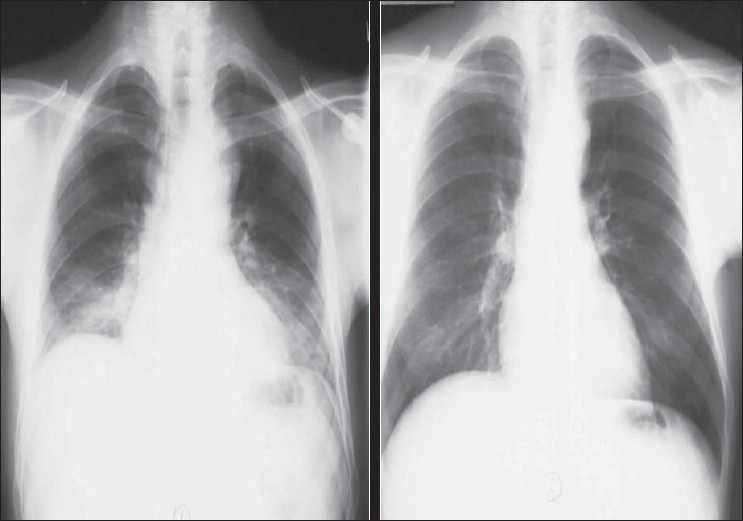
Chest radiographs on the same patient a few minutes apart showing the effect of technique; the left image shows mediastinal widening and basal clouding due to a poor inspiratory effort; the right image has been taken in good inspiration and looks entirely normal

The standard PA chest radiograph is rarely taken in the ICU patient and is replaced by an anteroposterior (AP) radiograph. These radiographs are ideally obtained in the AP projection with a patient–to–x–ray plate distance of 72 inches with the patient in the upright position at maximum inspiration; but more often, a distance of 40 inches is used in the supine or sitting position due to the impaired mobility of ICU patients [Figure 1]. A radiograph obtained in this way magnifies the mediastinum and heart due to gravitational and geometrical effects. Moreover, supine position alters the physiology of the pulmonary vasculature, diverting the blood to lung apices — an appearance that is regarded as normal on AP supine radiograph but considered abnormal on a PA radiograph. Supine radiographs have further limitations, including problems of differentiating pleural effusions from air space shadowing and detecting a pneumothorax. Exposing a radiograph in full inspiration in an ICU patient poses further challenges as these patients are often uncooperative or are suffering from postoperative pain [Figure 2]. A more-than-perfect inspiratory effort creates artifacts, making the diagnosis of basilar atelectasis and pulmonary edema difficult, besides causing changes in the apparent size of the heart and mediastinum.[1–4]

The sensitivity and specificity of the ICU chest radiograph are low, but its common use stems from studies that have shown that as much as 65% of ICU chest radiographs reveal a significant pathology that results in a change in patient management. Current recommendations from the American College of Radiology suggest that daily chest radiographs be obtained on patients with acute cardiopulmonary problems and those receiving mechanical ventilation. The college further recommends that only an initial chest radiograph is needed for the placement or change of indwelling devices.[5]

**Figure 3:**
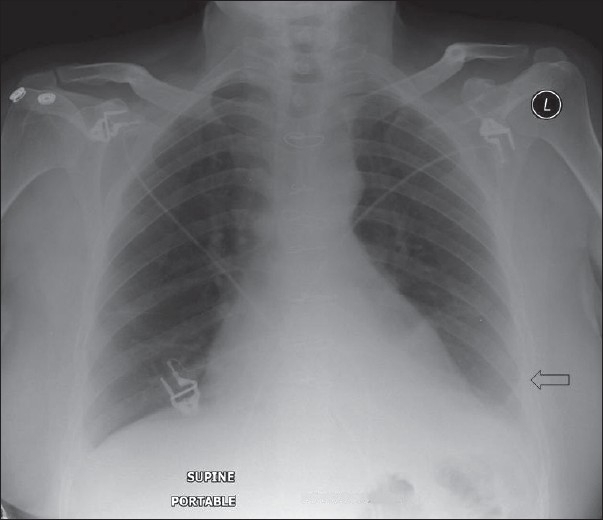
An AP chest radiograph on a 55-year-old female immediately following CABG, shows hazy opacification at the lung base and blunting of the left costophrenic angle due to a small pleural effusion, which is a common feature following thoracotomy (black arrow)

In patients that have undergone thoracotomy for whatever reason, the initial postoperative radiographs after return from operating theatre will show the lines and tubes placed perioperatively, such as an endotracheal tube, thoracostomy tubes, mediastinal drains and central venous catheters. These devices need to be identified and their positions checked for incorrect placement. Immediately following and a few days after CABG, lower lobe atelectasis is common, usually more pronounced on the left, generally resolving within a few days without complications. There is slight widening of the mediastinum, but significant increase in diameter might indicate complications such as a mediastinal bleeding. A small left pleural effusion is invariable following CABG, but a larger pleural effusion or a subsequent increase in size may need intervention so as not to cause respiratory compromise [Figure 3]. Therefore, comparison with previous radiographs is essential to assess a change in size of a pleural effusion.

## Identifying Lines and Tubes and Other Devices

**Figure 4:**
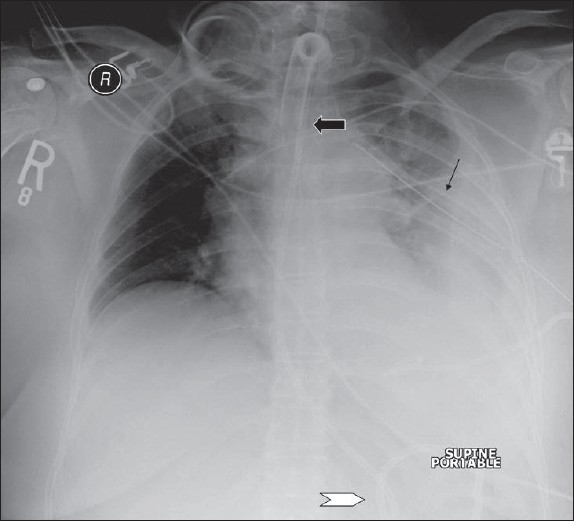
On return of patient from the operating theatre or following resuscitation, all tubes and lines should be checked and accounted for. In this patient, the position of the tracheostomy tube is satisfactory (black arrow), but the nasogastric tube is curled on itself and lies in the gastric fundus (white arrow); and the chest drain is also incorrectly placed for draining the pleural effusion (thin black arrow)

**Figure 5:**
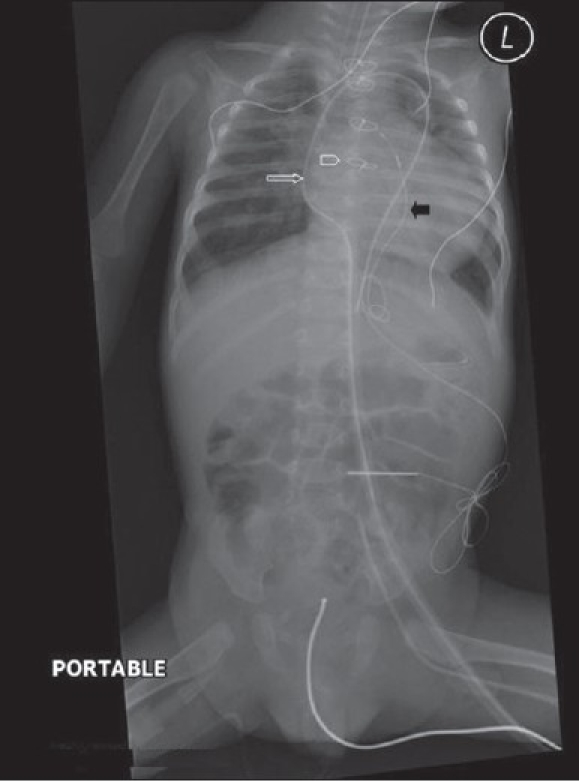
This child has had a midline sternotomy (hollow arrowhead); a mediastinal drainage catheter is seen in place (white hollow arrow); there is a pericardial pacing wire (black arrow) and a nasogastric tube — all in a satisfactory position

Tubes, lines and drainage catheters play a vital role in monitoring and treating critically ill patients. Accurate placement of these devices and monitoring malfunction are crucial. The initial portable chest radiograph plays an essential role in recognizing correct placement and complications. All placed devices should be identified on the preliminary radiograph as a priority in these patients before looking for cardiopulmonary disorders [Figures 4 and 5]. Table 1 summarizes the correct positioning of various lines and tubes.

**Table 1:** Identifying lines and tubes and other devices

**Endotracheal tubes**
Safe level 5 cm from carina (T4-T5 interspace), minimum distance 2 cm
**Nasogastric tube**
Ideally the distal duodenum
**CVP lines**
Ideally placed between proximal venous valves of the subclavian or jugular veins and the right atrium. Jugular venous placement has lower complications.
**Swan ganz catheter**
The tip is wedged into the distal pulmonary artery.
The balloon is deflated once the pressure is taken, and the tip is pulled back to the main pulmonary artery.
The tip of the catheter located within the mediastinal shadow indicates correct placement.
**The thoracostomy tube**
The last side-hole in a thoracostomy tube can be identified by an interruption in the radiopaque line.
This interruption in the radiopaque line should lie within the thoracic cavity, if not and or with evidence of subcutaneous air, a misplaced tube is suspected.
Incorrectly placed tubes for empyemas may delay drainage and result in loculation of the purulent fluid.
Thoracostomy tubes placed within pleural fissures often cease to drain when the lung surfaces become apposed.
**Cardiac pacemakers**
The tip of the cardiac pacemaker should be at the apex of the heart, and there should be no sharp angulations along the length of the pacemaker wires.
The lateral radiograph should show the tip imbedded within the cardiac trabeculae.
For correct placement to have occurred, the tip should appear 3 to 4 mm beneath the epicardial fat pad.
A tip that appears to be placed beyond the epicardial fat stripe may have perforated the myocardium.
Cardiac pacers placed within the coronary sinus appear to be directed posteriorly on the lateral chest radiographs.

### The endotracheal tube

**Figure 6:**
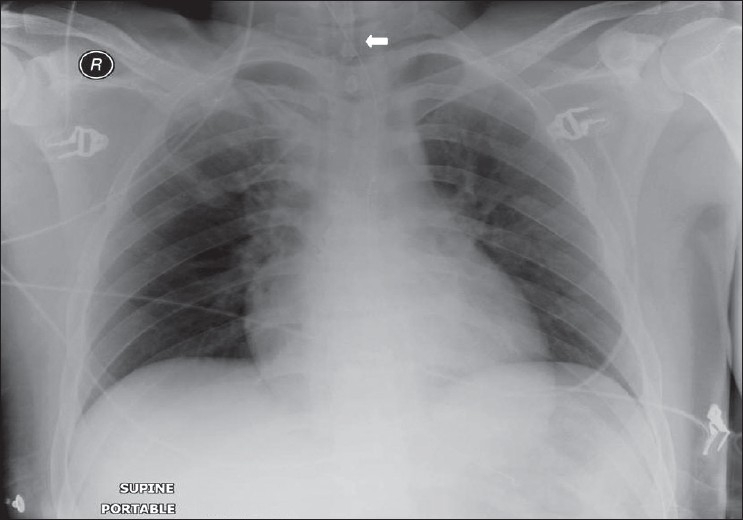
A position of the tip of the endotracheal tube is high at the level of the spinous process of D1 (arrow)

**Figure 7:**
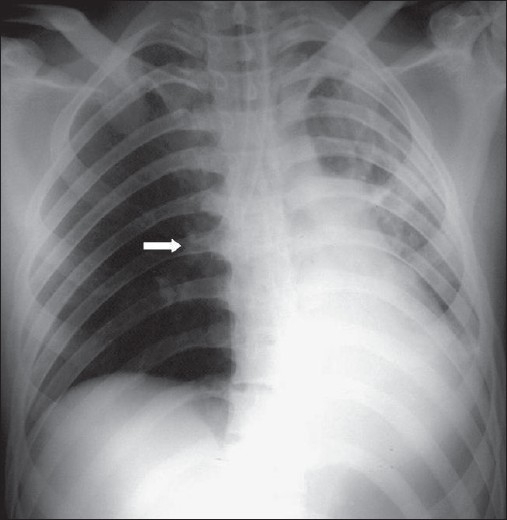
An incorrectly placed ET with the tip in the right main bronchus (arrow), causing partial atelectasis of the left lung

Endotracheal or tracheostomy tubes (ETs) maintain an airway access and allow mechanical ventilation of patients with respiratory failure. These tubes are cuffed and placed in the trachea, either via the oropharynx or introduced surgically through a tracheostomy. If it is anticipated that the patient needs intubation for a period longer than a week or has upper airway obstruction, a tracheostomy is usually preferred. A chest radiograph is essential to correctly locate the tip of endotracheal tubes. Misplaced ET may cause serious compromise of respiratory function and has been reported in 10% of the patients. Thus, daily chest radiographs are recommended in these patients as these devices may migrate. A correctly placed endotracheal tube lies at the level of the mid-trachea, about 5 cm from the carina. Placing the tip at this level allows for flexion or extension of the head. The minimal safe distance from the carina is 2 cm. Poor chest x-ray exposure may sometimes not allow recognition of the carina. A previous radiograph, if available, may be used to estimate the position of the carina. Alternatively, the position of the tip of the ET can be assessed by looking at the upper dorsal spine. As the carina normally lies at the T4-T5 interspace, the tip of the endotracheal tube lying at this level is regarded as correct placement [Figures 6 and 7]. The Dee method has been devised for approximating the position of the carina. This involves identifying the aortic arch and then drawing a line inferomedially through the middle of the arch at a 45-degree angle to the midline. The intersection of the midline and the diagonal line is the most likely position of the carina. This is a cumbersome method for the busy ICU physician/radiologist and is seldom used.

ETs are misplaced in approximately 10% of the patients. A comparatively common misplacement is when the tube enters the right main stem bronchus, due to its more vertical orientation. This position impairs the left lung ventilation, leading to collapse of the left lung; similarly, if the endotracheal tube enters the bronchus intermedius, the right upper lobe may collapse. ET that has been placed at a higher level may slip into the pharynx or the esophagus, causing gastric air distension with the potential danger of reflux of gastric contents and aspiration. More serious complications of ETs include tracheal stenosis, tracheal rupture, cord paralysis, cervical and mediastinal emphysema, hematoma and abscess formation.

When upper airway injury is suspected, a lateral radiograph may be useful. The soft tissue space between the trachea and cervical spine is increased in diameter to over the width of one vertebral body in the presence of a hematoma or infection.

**Figure 8:**
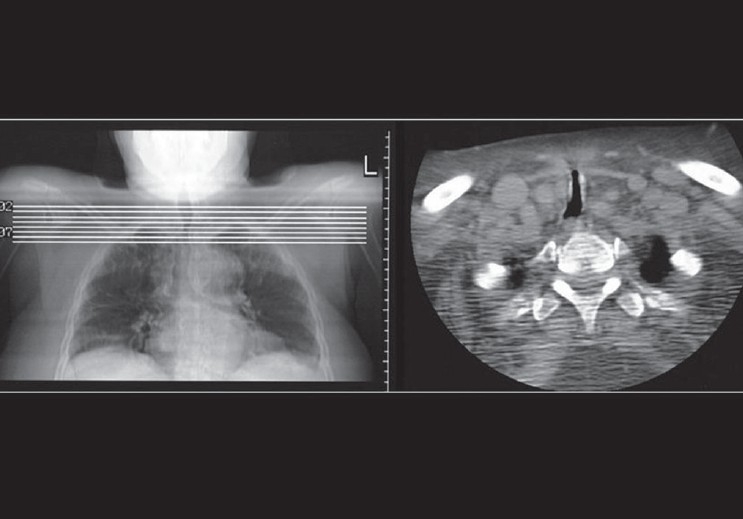
A scout film from a CT scan (left) shows narrowing of the trachea following prolonged ET placement. An axial CT confirms tracheal stenosis (right)

Severe injury such as tracheal rupture should be suspected in patients with pneumothorax, pneumomediastinum, subcutaneous emphysema in the neck or precipitous respiratory failure following intubation [Figure 8]. Most tracheal ruptures are placed posteriorly.[6–9]

### The thoracostomy tube

**Figure 9:**
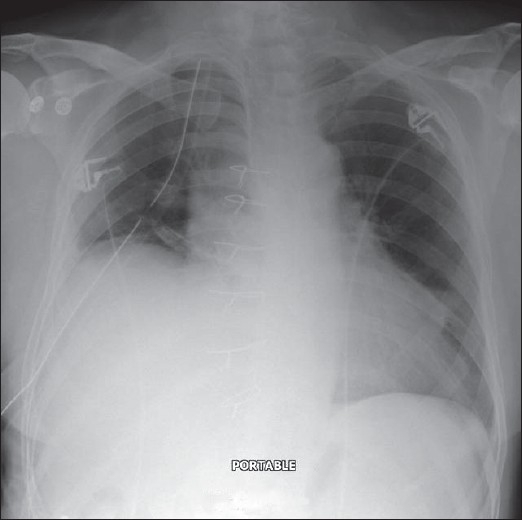
A subpulmonic effusion mimicking an elevated right hemidiaphragm. A pleural drain has been misplaced

Thoracostomy tubes are often placed into the pleural space to treat a pneumothorax or drain pleural fluid [Figure 9]. Chest radiographs are often obtained following placement of thoracostomy tubes to identify their position. It is important to recognize that on a supine AP radiograph, air accumulates anteriorly and fluid gravitates posteriorly. This fact is taken into account when placing thoracostomy tubes relevant to the pathology in hand. Determining whether a tube is anterior or posterior is often difficult with a single AP chest radiograph. Thoracostomy tubes placed within pleural fissures often cease to drain when the lung surfaces become apposed. Correct placement of thoracostomy tube fenestrations within the thoracic cavity is important to proper functioning of these tubes. The last side-hole in a thoracostomy tube can be identified by an interruption in the radiopaque line. This interruption in the radiopaque line should lie within the thoracic cavity, if not and or with evidence of subcutaneous air, a misplaced tube should be suspected. Incorrectly placed tubes for empyemas may delay drainage and result in loculation of the purulent fluid.[10]

### The feeding tube

**Figure 10:**
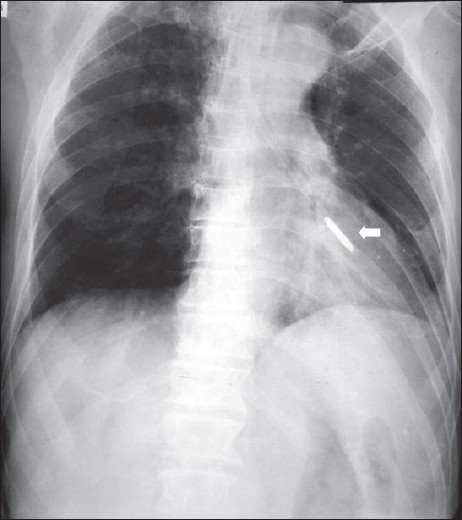
The nasogastric tube has entered the left lower lobe bronchus, causing partial collapse and consolidation of the left lower lobe. This serious misplacement can particularly happen in unconscious patients and patients on ventilators

**Figure 11:**
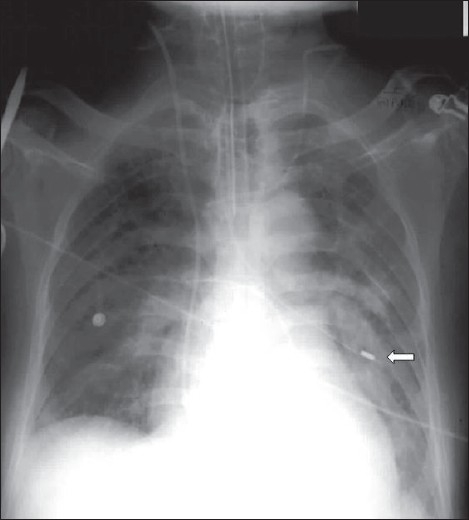
A small-bore feeding tube has been misplaced into the left lower lobe bronchus, causing left lower lobe consolidation (solid white arrow)

**Figure 12:**
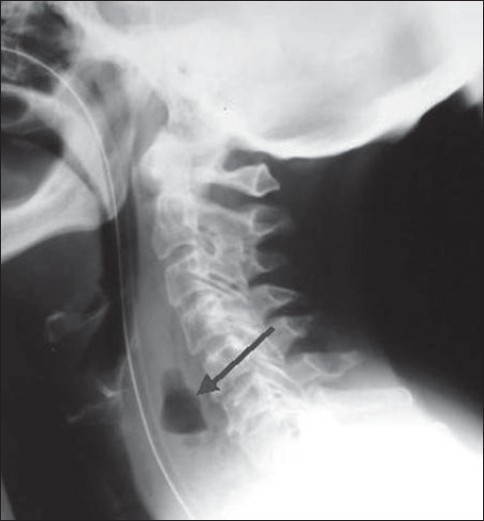
A lateral radiograph of the neck showing a collection of air in the prevertebral soft tissue space due to esophageal perforation secondary to a difficult intubation (arrow)

Nasogastric tubes (NGs) are used to feed patients or for gastric aspiration in appropriate clinical circumstances. Radiographs are seldom required for accurate placement of NG tubes and are not used except when the patient is unconscious and there is risk of placement of the tube into the bronchial tree. There are other exceptions where a chest radiograph is useful and may prevent serious consequences, which include instances where small-bore feeding tubes are inserted and in status-post esophagectomy [Figures 10–12]. The lower tip of the NG is generally placed in the upper small bowel, which may be confirmed with an abdominal radiograph.[11–14]

### Monitoring central venous pressures

**Figure 13:**
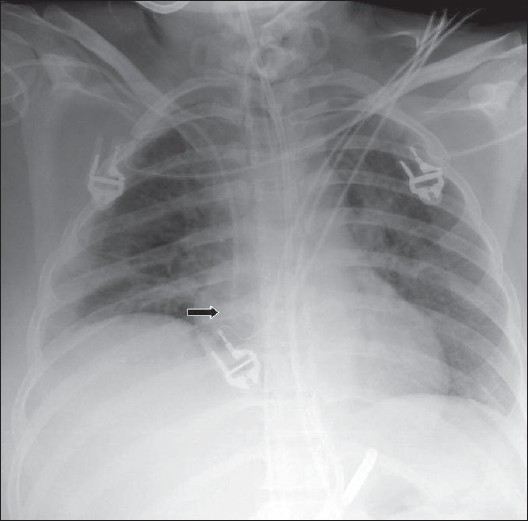
AP chest radiograph showing the tip of an intravenous line within the left atrium (arrow)

**Figure 14:**
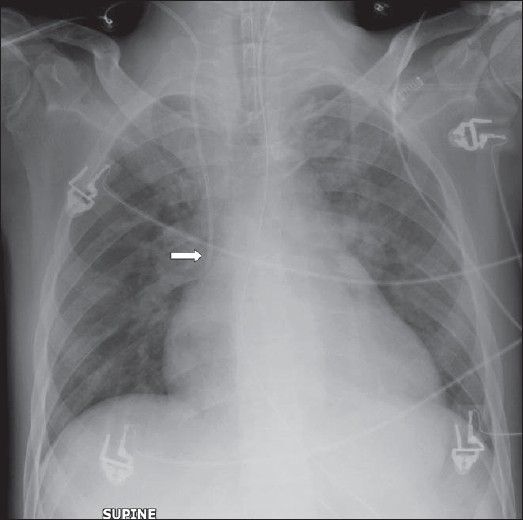
Radiograph showing the tip of an intravenous line to lie at the junction of the superior vena cava and the left atrium

**Figure 15:**
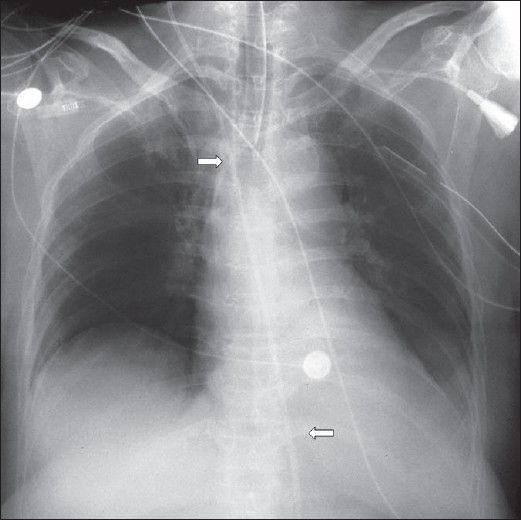
The intravenous line has crossed over from the superior vena cava into the azygos vein (arrows)

**Figure 16:**
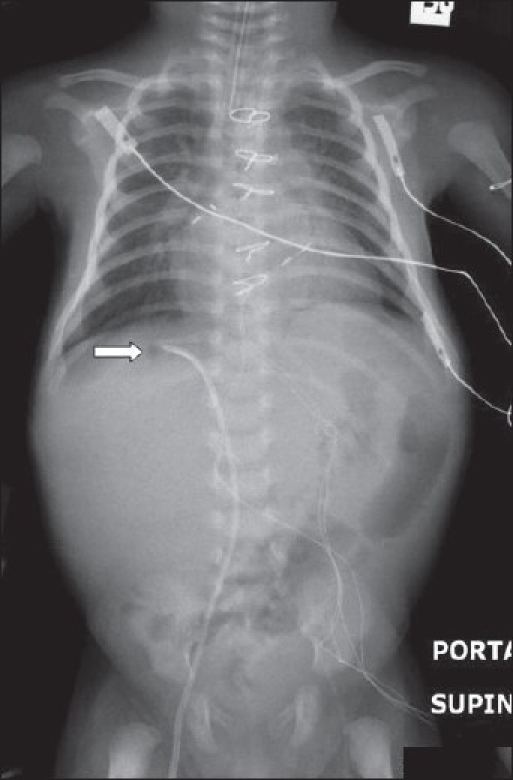
Radiograph of chest and abdomen of a neonate following cardiac surgery, showing a misplaced intravenous line into a hepatic vein. The intravenous line has been introduced via the right femoral vein

**Figure 17:**
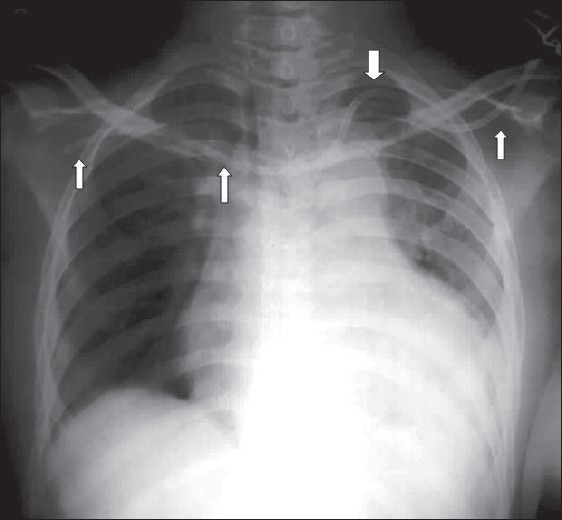
A misplaced large-bore intravenous line crossing from the left subclavian vein into the right subclavian vein

Central venous pressures (CVPs) are monitored by central vein catheters placed either through the subclavian or the internal jugular vein; or occasionally via the femoral vein, particularly in babies where access via jugular or subclavian vein is unavailable. These catheters are also used for safe delivery of large volumes of fluids over long periods with minimal chances of venous thrombosis. Correct placement of the tip of the CVP line is important for accurate measurement of central venous pressure. The ideal location of the tip of the CVP line is between the most proximal venous valves of the subclavian or jugular veins and the right atrium. Misplacing CVP lines is not uncommon when lines are placed within the internal jugular vein, right atrium and right ventricle. Placing the CVP catheter distal to the superior vena cava may cause arrhythmias or may risk cardiac a perforation [Figures 13–17]. Other complications of CVP line placement are a pneumothorax; and intimal injury to veins, causing perforation or thrombosis. These complications can be avoided by using ultrasound guidance for CVP catheter placement, and jugular vein placement is preferred because of lower complication rates.[15–17]

### Monitoring pulmonary capillary wedge pressure

**Figure 18:**
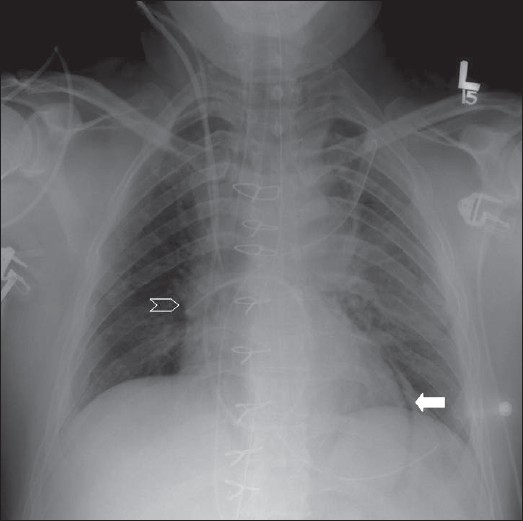
Left pneumopericardium (solid white arrow). Note that the JVP line is also falling short of the SVC (hollow white arrow). The tip of Swan-Ganz catheter lies within the right main pulmonary artery

**Figure 19:**
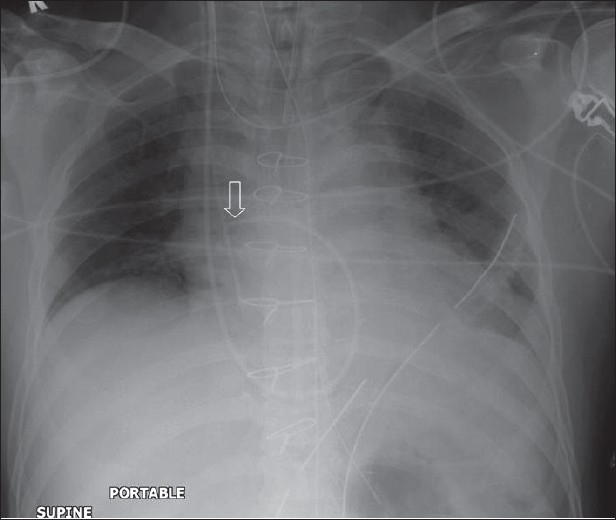
The tip of Swan-Ganz catheter has been withdrawn into the right main pulmonary artery (hollow arrow)

**Figure 20:**
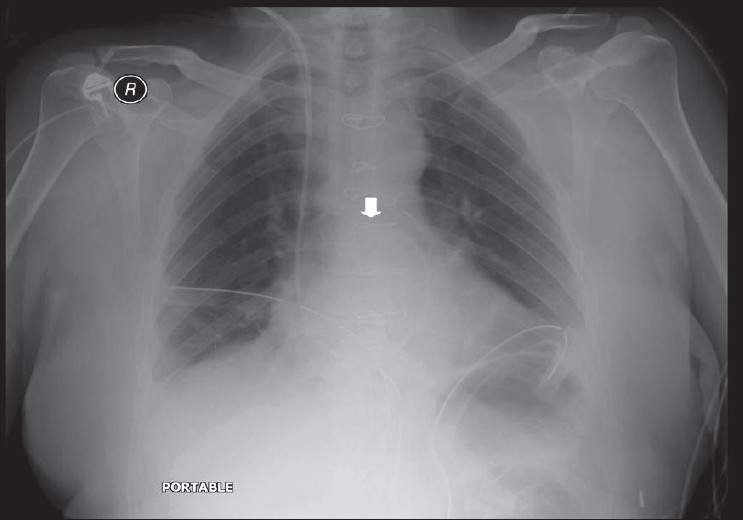
The tip of Swan-Ganz catheter has been withdrawn further into the main pulmonary artery (arrow)

Pulmonary capillary wedge pressure monitors are introduced via the venous system to help accurate assessment of the patient's volume status and can help differentiate between cardiac and noncardiac pulmonary edema. Swan-Ganz catheters are generally used as pulmonary capillary wedge pressure monitors. These catheters are introduced percutaneously via the right heart and into the pulmonary artery. This allows pulmonary wedge pressure to be calculated by inflating a balloon located at the tip of the catheter. The tip is advanced into a distal pulmonary artery and wedged there. The balloon is deflated once the pressure is taken, and the tip is pulled back to the main pulmonary artery. The tip of the catheter located within the mediastinal shadow indicates correct placement. The catheter tip should ideally be placed proximal to an interlobar pulmonary artery [Figures 18–20]. Malpositioning of Swan-Ganz catheters may occur in a quarter of the patients, resulting in false pulmonary capillary wedge pressure readings, risk for pulmonary infarction, pulmonary artery perforation, cardiac arrhythmias and endocarditis.[18–23]

### The intra-aortic counterpulsation balloon pump

**Figure 21:**
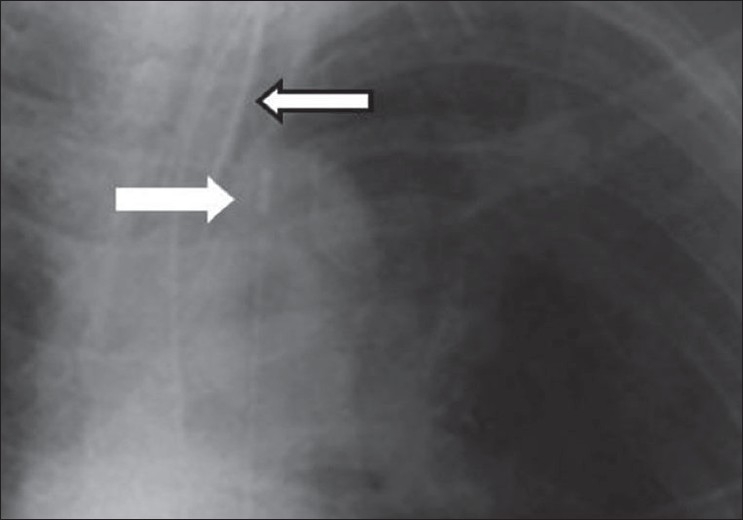
Magnified view of the aortic knuckle showing the tip of an intra-aortic balloon pump (solid white arrow); the endotracheal tube is marked by the hollow arrow

The intra-aortic counterpulsation balloon pump (IACB) is used to decrease afterload and increase cardiac perfusion in patients with cardiogenic shock. The device is synchronized with either the aortic pressures or the patient's EKG, to inflate during diastole and deflate during systole [Figure 21]. The device is introduced via the right femoral artery to be positioned above the celiac axis in the region of the aortic isthmus or left main bronchus. During systole, the lucent air-filled balloon appears as fusiform.[24–26]

### Cardiac pacing devices

**Figure 22:**
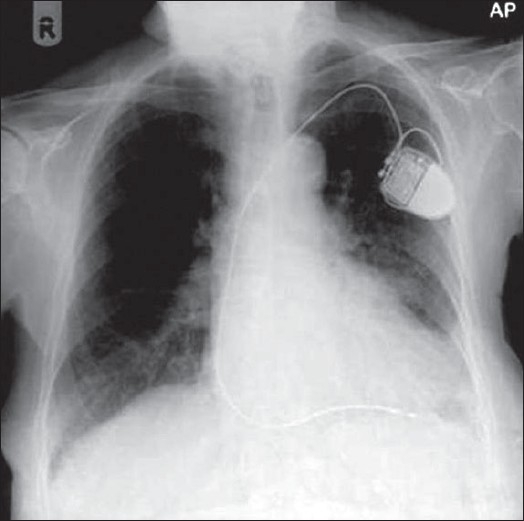
A check radiograph following placement of a cardiac pacemaker shows the position of electrode to lie within the apex of the right ventricle

**Figure 23:**
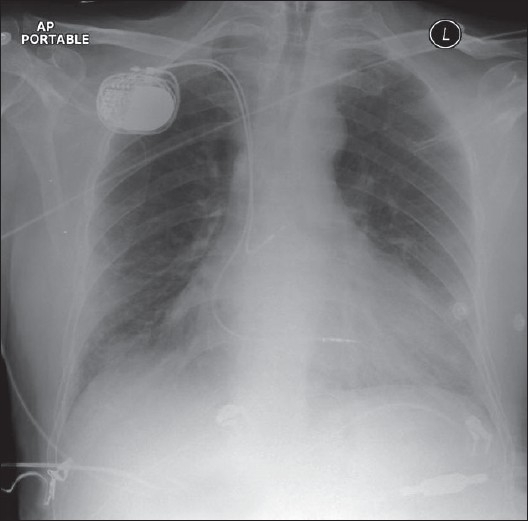
A dual-lead cardiac pacemaker is seen *in situ*; the ventricular lead falls short of the apex of the right ventricle

**Figure 24:**
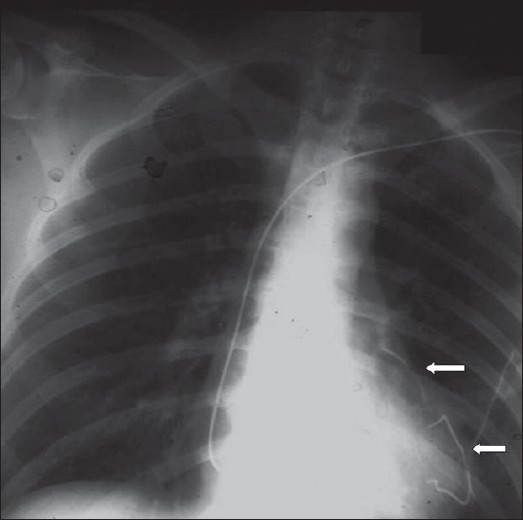
Fractured pacemaker wires; pieces lie in the right ventricle (lower arrow) and the left lobe pulmonary artery (upper arrow)

ICU patients with cardiac arrhythmias or a heart block may require temporary cardiac pacemakers. The pacing wires of these devices are introduced via the cephalic or subclavian vein into the apex of the right ventricle. AP and lateral chest radiographs are usually required to evaluate accurate pacemaker placement. The tip of the cardiac pacemaker should be at the apex of the heart, and there should be no sharp angulations along the length of the pacemaker wires. The lateral radiograph should show the tip imbedded within the cardiac trabeculae. For correct placement to have occurred, the tip should appear 3 to 4 mm beneath the epicardial fat pad [Figures 22–24]. A tip that appears to be placed beyond the epicardial fat stripe may have perforated the myocardium. Cardiac pacers placed within the coronary sinus appear to be directed posteriorly on the lateral chest radiographs.[27–30]

### Pneumothorax and other intrathoracic air collections

Extra-alveolar thoracic air collections in the ICU patients are not uncommon — often the result of intubation, other thoracic interventional procedures and barotrauma from positive end-expiratory pressure ventilation. This extra-alveolar air can collect as pulmonary interstitial emphysema, pneumothorax, pneumomediastinum, pneumopericardium or subcutaneous air.

**Figure 25:**
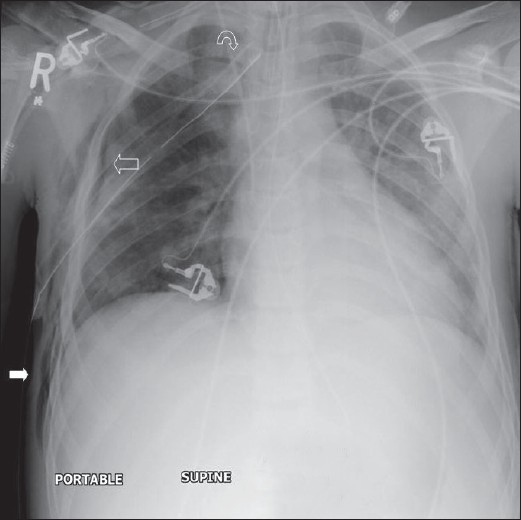
A portal supine chest radiograph showing surgical emphysema (solid white arrow) following placement of chest drain (curved arrow). Note the shallow pneumothorax (hollow white arrow). Surgical emphysema makes diagnosis of a pneumothorax difficult

Subcutaneous emphysema is not uncommon and often follows percutaneous intrathoracic placement of drains and other devices. Air dissects through the fascial planes, which usually has no clinical consequences [Figure 25]. Subcutaneous emphysema may be associated with a pneumomediastinum; however, in the presence of isolated cervical subcutaneous emphysema, the patient needs to be examined for upper airway injury, particularly relevant following a difficult intubation or the placement of a new nasogastric tube. The chest x-ray findings of subcutaneous emphysema may be striking; and the air dissecting the fascial planes, particularly between muscle bundles in the pectoralis region, is well seen obscuring underlying lung parenchymal changes. In the presence of subcutaneous emphysema, the detection of a pneumothorax also can become difficult.

### Pneumothorax

**Figure 26:**
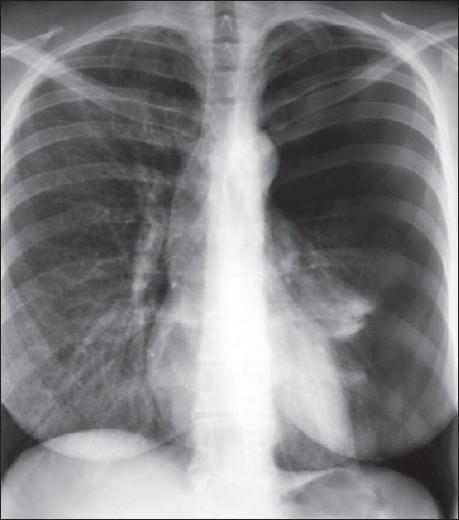
A frontal chest radiograph showing a large left side pneumothorax causing almost complete collapse of the left lung

**Figure 27:**
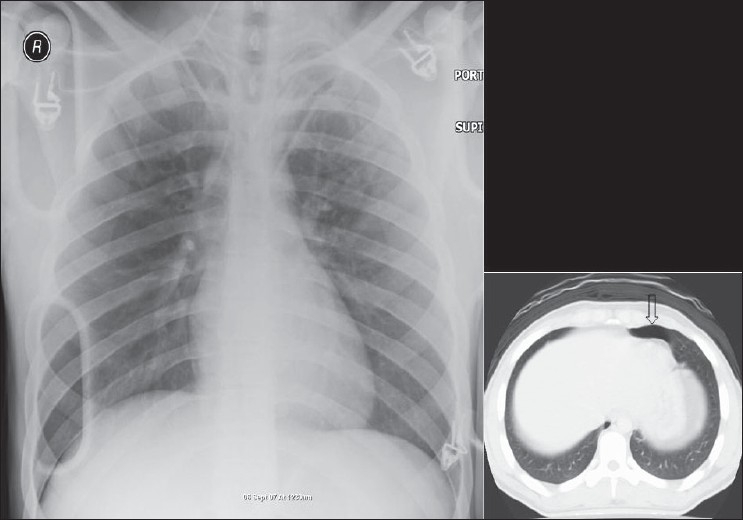
AP chest radiograph may miss a small pneumothorax. One such example is seen above; note the small collection of air anteriorly on the axial CT, representing a small pneumothorax

**Figure 28:**
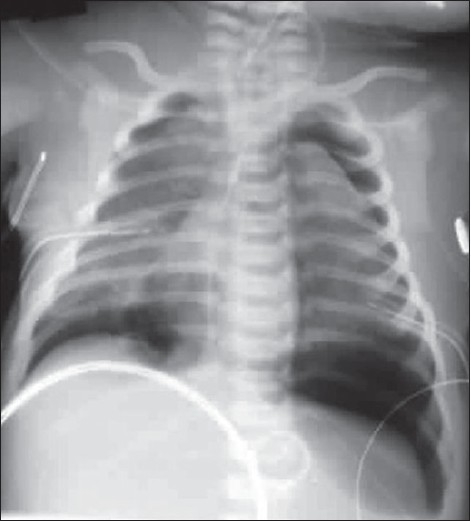
Bilateral pneumothorax seen in the neonate with meconium aspiration

**Figure 29:**
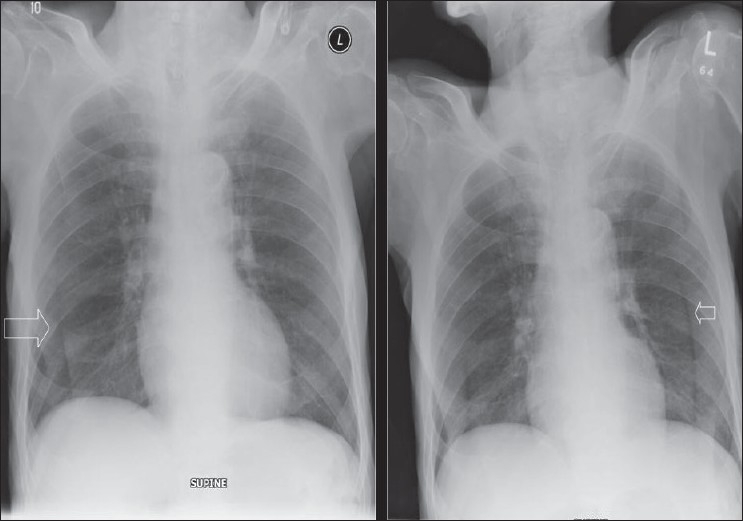
Multiple skin folds mimicking a pneumothorax (arrows)

A pneumothorax represents accumulation of air in the pleural space and it may occur spontaneously, or secondary to trauma, it may or may not be associated with lung parenchymal disease. Air rises to the nondependent position, and the radiographic appearance depends upon how the radiograph has been exposed. In the erect patient, air rises to apicolateral surface of the lung and appears as a thin, white pleural line with no lung markings beyond [Figures 26–28]; however, the presence of lung markings beyond the pleural line does not exclude a pneumothorax. The diagnosis of a pneumothorax may be particularly difficult in the presence of parenchymal disease, which may alter the compliance or affect the compliance of the lung, making collapse more difficult. A skin fold may mimic a pneumothorax. A skin fold line when followed continues outside of the chest [Figure 29].

In an ICU patient, diagnosis of pneumothorax is often made on a supine radiograph. In a supine patient air rises anteromedially. An apical air collection in a supine patient is a sign of a large pneumothorax. Air can sometimes be trapped in a subpulmonic location between the lung and the diaphragm. Anterolateral extension of air into the costophrenic sulcus may increase the radiolucency at the costophrenic sulcus. This is called the deep sulcus sign. Other features of a subpulmonic pneumothorax include visualization of the superior surface of the diaphragm and the superior part of the inferior vena cava.

A tension pneumothorax is accumulation of air within the pleural space due to free ingress of air with limited egress of air. The intrapleural pressure exceeds atmospheric pressure in the lung during expiration (ball-valve mechanism). The diagnosis of tension pneumothorax is a clinical one based on respiratory and cardiac compromise. Diagnosis of a tension pneumothorax in a critically ill patient can be extremely challenging, partly due to the fact that lung pathology such as ARDS may reduce lung compliance, preventing total lung collapse as occurs in a tension pneumothorax. Similarly, a mediastinal shift, a hallmark of tension pneumothorax, may not occur with the use of PEEP. Signs of a tension pneumothorax include depression of a hemidiaphragm, a shift of the heart border, the superior vena cava and the inferior vena cava.[31–32]

### Pneumomediastinum

**Figure 30:**
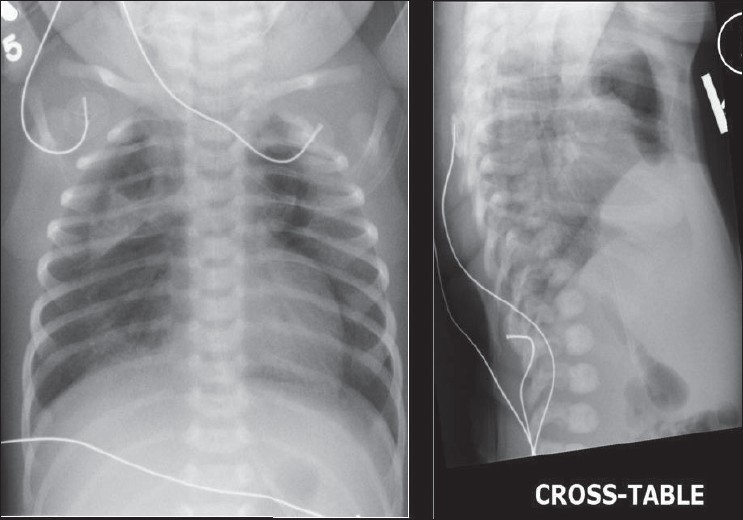
AP and cross-table chest radiograph on a 1-year-old child showing extensive pneumomediastinum; note that the air is extending to the root of the neck, differentiating it from a pneumopericardium)

**Figure 31:**
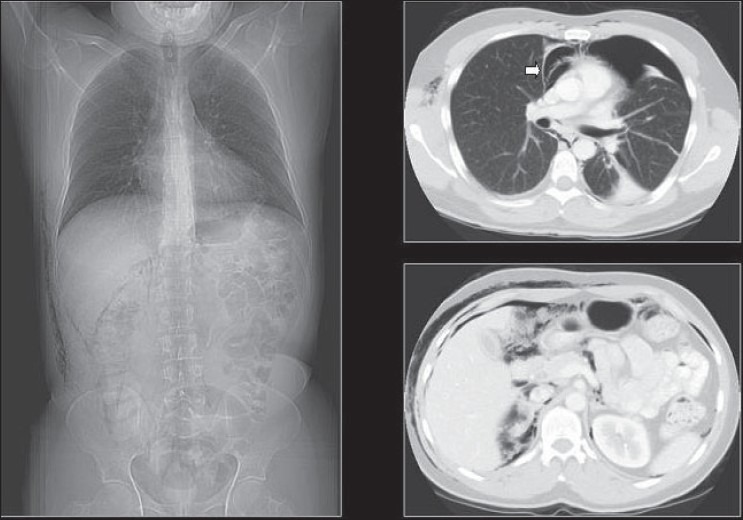
A scout film and axial CT scans showing the distribution of air following a retropneumoperitoneum. Note the pneumomediastinum (arrow)

A pneumomediastinum represents air in the mediastinum and may be related to pulmonary interstitial air dissecting centripetally in the intubated patient [Figures 30 and 31]. Mediastinal air may also represent a leak from a major airway injury or air dissecting through fascial planes from the retroperitoneum. Unlike a pneumopericardium, air from a pneumomediastinum often dissects up into the neck. Moreover, a pneumopericardium can extend inferior to the heart. A pneumomediastinum usually remains asymptomatic. However, occasionally a retrosternal crunch may be heard on auscultation. Radiographic features of a pneumomediastinum include air around the great vessels, the medial border of the superior vena cava, and the azygos vein seen as surrounding lucencies. Air may also be seen outlining the aortic knuckle, descending aorta and the pulmonary arteries. A posteromedial pneumomediastinum is usually the result of esophageal rupture, where air dissects into the paraspinal costophrenic angle and beneath the parietal pleura of the left diaphragm. The result is a V-shaped lucency called the V-sign of Naclerio.[33]

### Pneumopericardium

**Figure 32:**
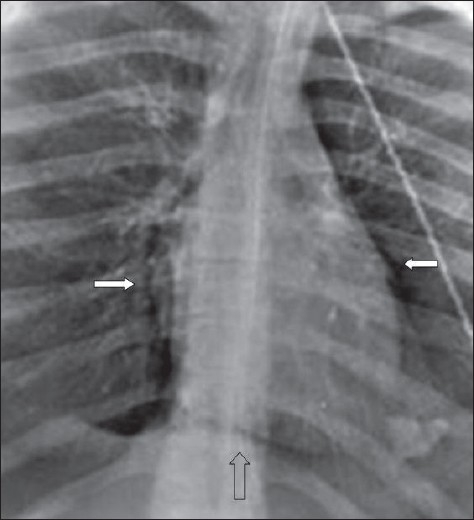
Chest x-ray findings of a pneumopericardium shown as a lucent line around the heart extending up to the main pulmonary arteries (solid white arrows). Air may accumulate inferior to the cardiac shadow, which crosses the midline above the diaphragm, which is said to be diagnostic for pneumopericardium, the so-called continuous diaphragm sign (hollow arrow)

**Figure 33:**
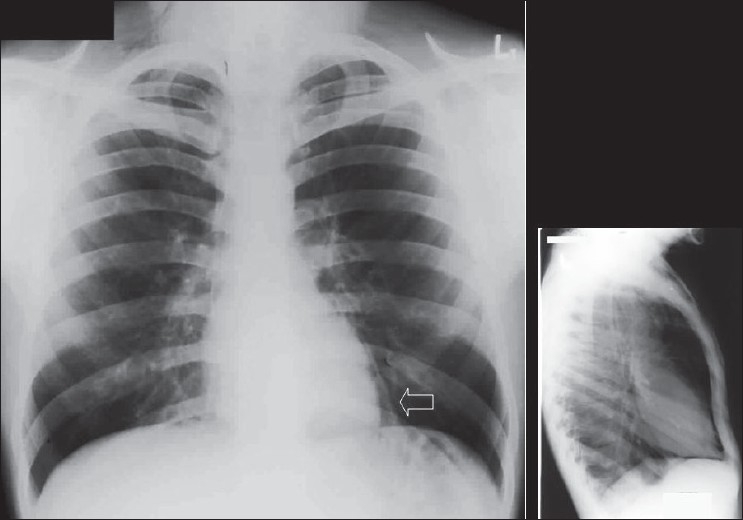
Frontal and lateral radiographs depicting a pneumopericardium

**Figure 34:**
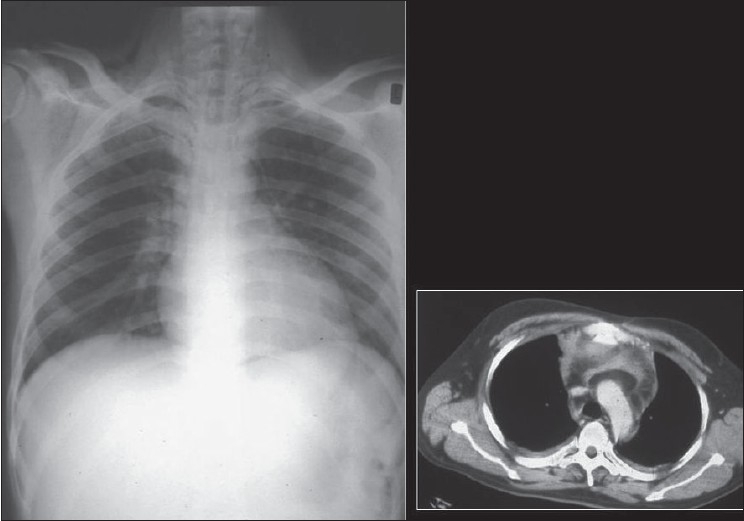
A frontal radiograph and axial CT depicting a pneumopericardium. Note the surgical emphysema at the root of the neck

A pneumopericardium refers to an accumulation of gas/air between the myocardium and pericardium [Figures 32–34]. Pneumopericardium can be an occasional complication of pneumothorax but is more often found in the postoperative cardiac patient. The chest x-ray findings are those of a lucent line around the heart, extending up to the main pulmonary arteries. Air may accumulate inferior to the cardiac shadow, which crosses the midline above the diaphragm, which is said to be diagnostic for pneumopericardium, the so-called continuous diaphragm sign.[34]

### Pleural effusions

**Figure 35:**
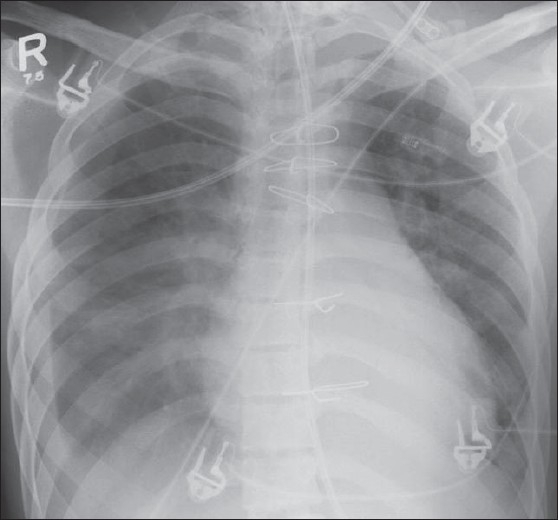
Hazy opacification of the whole of the left hemithorax, suggestive of pleural effusion following thoracic surgery

**Figure 36:**
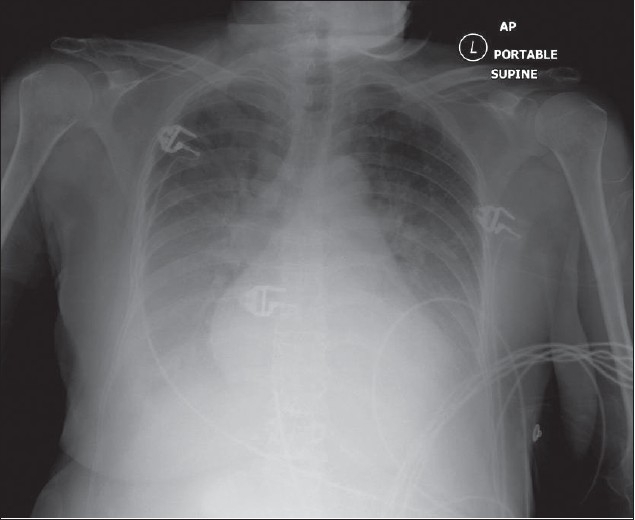
Bilateral pleural effusions following fluid overload. Note the bilateral basal and mid-zone opacification and obscuration of the hemidiaphragms. The lack of an air bronchogram excludes air space consolidation

**Figure 37:**
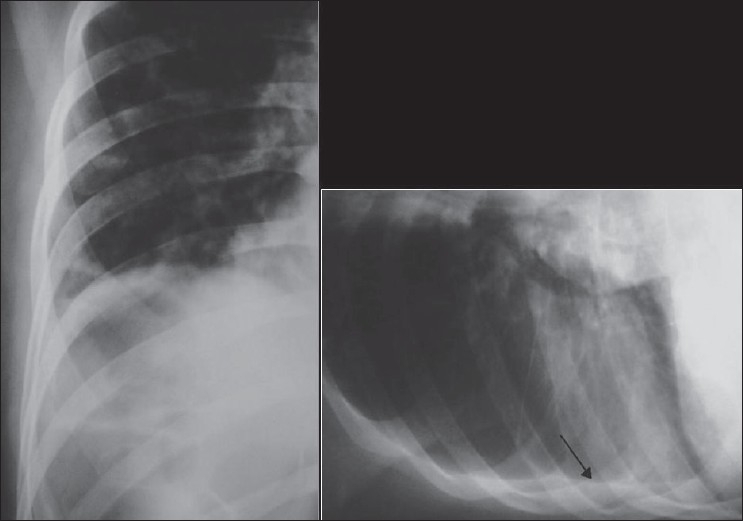
Radiographs showing the value of lateral radiography in the detection of smaller pleural effusions. There is blunting of the right costophrenic angle on the erect radiograph (left), but the appearances are not diagnostic of a pleural effusion. However, the lateral decubitus film (right) shows layering of the pleural fluid (arrow)

**Figure 38:**
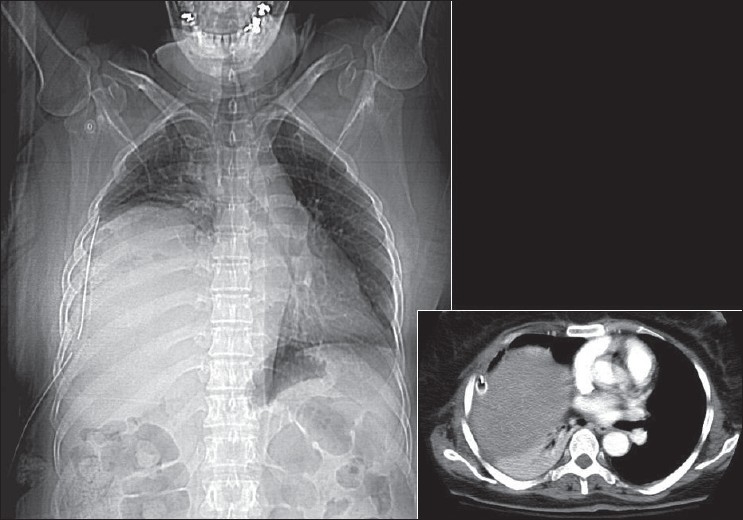
A CT scout film shows a subpulmonic effusion with a misplaced pleural drain. The axial scan confirms the presence of a subpulmonic effusion and depicts the misplaced pleural drain

**Figure 39:**
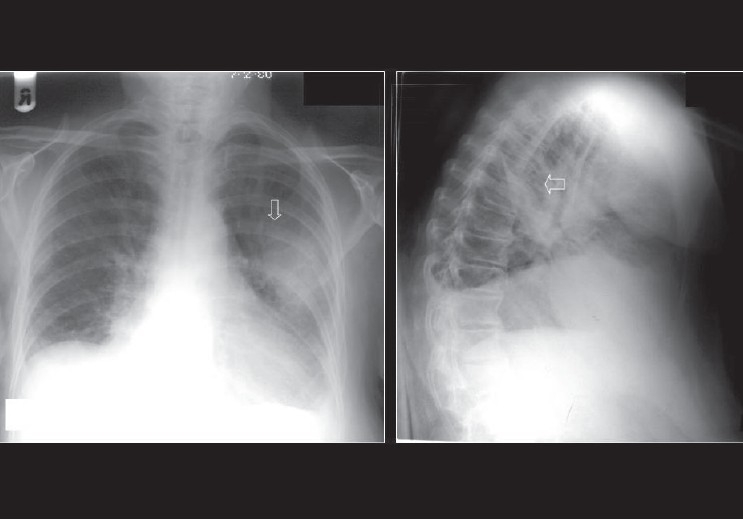
Encysted pleural effusion seen en face as an oval opacity; its margin is partially well defined and partially ill defined (AP radiograph). On the lateral radiograph, the effusion appears as a homogenous density with biconvex edges

**Figure 40:**
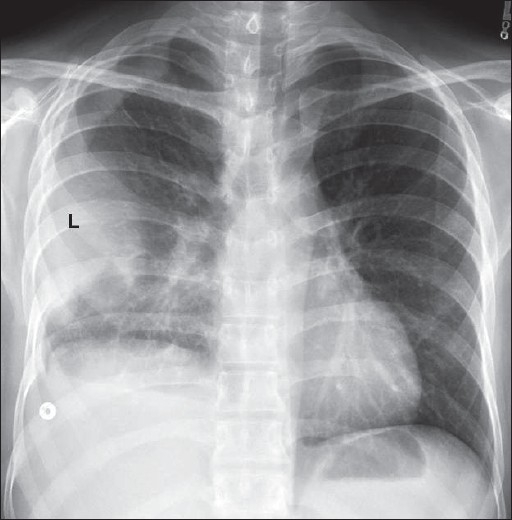
Loculated pleural effusion along the left lateral chest wall, which mimics an extra-pleural mass (L), but hazy opacification at the right lung base and blunting of the right costophrenic angle suggest pleural disease/thickening

**Figure 41:**
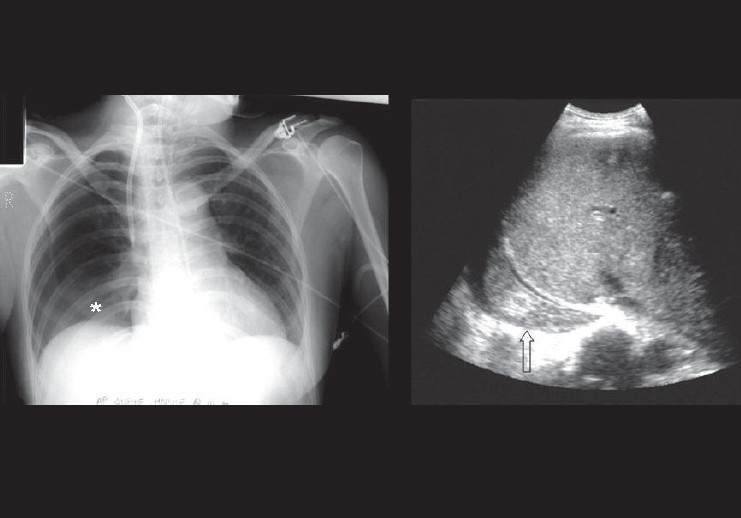
Frontal radiograph showing vague opacification at the left lung base, suggestive of a pleural effusion that followed a difficult intravenous line placement. The ultrasound image (right) shows solid component within the posterior costophrenic angle, suggestive of a hemothorax. An ultrasound scan can easily differentiate a clear pleural effusion from a hemorrhagic pleural effusion

Pleural effusions are accumulations of fluid within the pleural space [Figures 35–41]. Pleural effusions occur frequently in the ICU patients, which may be secondary to heart failure, fluid overload, hypoproteinemia, infection, pulmonary embolism, thoracic and upper abdominal surgery, neoplastic disease, subphrenic inflammatory processes, trauma and ascites. The fluid could be blood, chyme, pus, transudates or exudates. The radiographic appearance of a pleural effusion is dependent on the position of the patient. Pleural fluid accumulates in the dependent areas of the chest. A pleural effusion is easier to identify in the erect patient as fluid collects at the base of the lung, causing costophrenic angle blunting and decreased visibility of the lower lobe vessels. In the supine position, identification of a pleural effusion is more challenging. In the supine position, pleural fluid accumulates in the posterior basilar space, which appears as homogenous density that increases in intensity towards the lung base. The normal bronchovascular markings are retained in this veil-like density. With increasing amount of pleural fluid, the diaphragm loses its contour and costophrenic angle may be obliterated. However, it should be remembered that the pleural space may accommodate up to a liter of fluid above the diaphragm without blunting of the costophrenic angle. With larger pleural effusions, the fluid may appear as pleural cap at the lung apex, making it easier to identify on a supine radiograph. The fluid may sometimes accumulate on the medial side of the lung, appearing as a widened mediastinum. Often, smaller pleural effusions are missed on supine chest radiographs despite meticulous technique. When effusions are not readily apparent on a supine chest radiograph but clinically suspected, a lateral decubitus film is indicated. The film should be taken with the side of the patient suspected to have an effusion in the dependent position. The lateral decubitus film would not only confirm smaller pleural effusions but can also differentiate between loculated and free effusions. The latter information is important when pleural drainage is planned, as loculated effusions may need more than one drain. A pleural effusion at the lung base is termed a subpulmonic effusion and is a common occurrence in the ICU patient. On the chest radiograph, a subpulmonic pleural effusion appears as a raised hemidiaphragm with flattening and lateral displacement of the dome. A lateral decubitus film can usually resolve this.

Loculated pleural effusions may pose a diagnostic challenge, especially when fluid is retained within the fissures; and in particular, when the fissures are incomplete. A loculated effusion in the minor fissure and right middle lobe atelectasis may be difficult to differentiate on a supine chest radiograph. Interlobar effusion appears as a homogenous density with biconvex edges and preservation of the minor fissure, while atelectasis appears as an inhomogeneous density with concave margins and obliteration of both the right heart border and minor fissure. CT or an erect lateral radiograph, if possible, may resolve the issue.[35–39]

### Pericardial effusions

**Figure 42:**
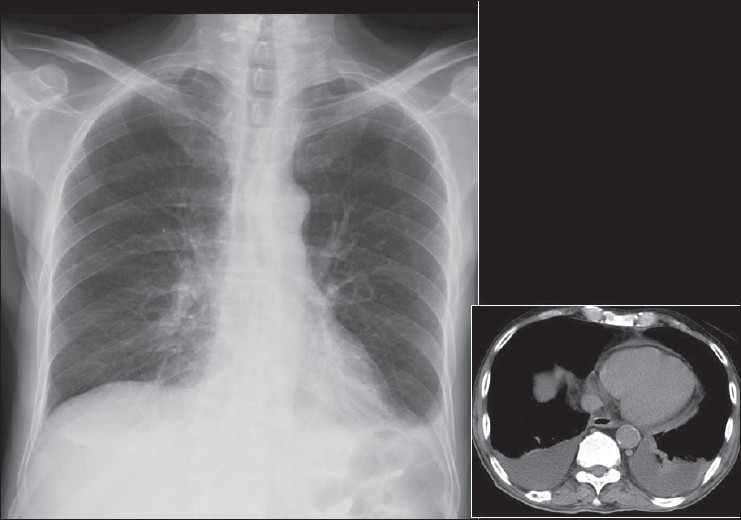
The chest radiograph and CT scan were taken on the same day, 7 hours apart. There is nothing suspicious on the chest radiograph to suggest a pericardial effusion. There is blunting of the left costophrenic angle, suggestive of a small pleural effusion. However, the axial CT scan shows a small pericardial effusion and moderate bilateral pleural effusions

**Figure 43:**
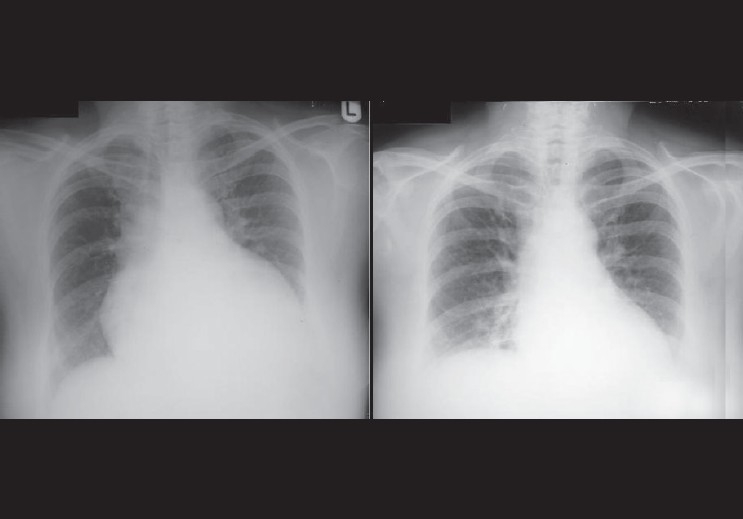
The radiograph on the left was taken on admission of the patient, showing an enlarged globular heart secondary to pericardial effusion due to severe hypothyroidism. The image on the right was taken 3 weeks later, showing resolution of the pericardial effusion

**Figure 44:**
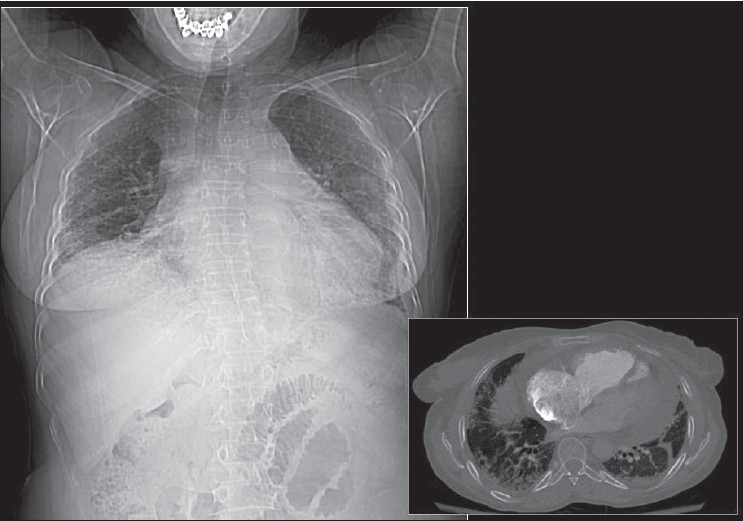
Pericardial effusions may not be that obvious on a chest radiograph. A CT scout film (left) shows nonspecific cardiomegaly in a patient with a clinical diagnosis of viral myocarditis. An axial CT section through the mediastinum shows a moderate-sized pericardial effusion

Pericardial effusions are accumulations of fluid between the visceral and parietal pericardium [Figures 42–44]. They usually cannot be seen on plain chest radiographs, and smaller effusions are difficult to differentiate from cardiomegaly. A variety of pathologies may cause pericardial effusions, including lymphatic or venous obstruction by tumors, changes in osmotic pressures, or inflammation of the pericardium causing increased permeability. Generally, pericardial effusions only become symptomatic when the intrapericardial pressure rises by 3 or 4 mm Hg. A hemopericardium may follow cardiac surgery or trauma. The rapidity at which the pericardial effusion accumulates dictates hemodynamic consequences. Radiographically, a pericardial effusion appears as cardiomegaly with a change in cardiac silhouette, resulting in a featureless, globular or “water bottle” shape. The best and quickest way to determine the presence of a pericardial effusion is by echocardiography.[40–43]
